# Characterization of the Major Light-Harvesting Complexes (LHCBM) of the Green Alga *Chlamydomonas reinhardtii*

**DOI:** 10.1371/journal.pone.0119211

**Published:** 2015-02-27

**Authors:** Alberto Natali, Roberta Croce

**Affiliations:** Department of Physics and Astronomy, Faculty of Sciences, VU University Amsterdam, De Boelelaan 1081, 1081 HV Amsterdam, The Netherlands; University of Hyderabad, INDIA

## Abstract

Nine genes (LHCBM1-9) encode the major light-harvesting system of *Chlamydomonas reinhardtii*. Transcriptomic and proteomic analyses have shown that those genes are all expressed albeit in different amounts and some of them only in certain conditions. However, little is known about the properties and specific functions of the individual gene products because they have never been isolated. Here we have purified several complexes from native membranes and/or we have reconstituted them *in vitro* with pigments extracted from *C. reinhardtii*. It is shown that LHCBM1 and -M2/7 represent more than half of the LHCBM population in the membrane. LHCBM2/7 forms homotrimers while LHCBM1 seems to be present in heterotrimers. Trimers containing only type I LHCBM (M3/4/6/8/9) were also observed. Despite their different roles, all complexes have very similar properties in terms of pigment content, organization, stability, absorption, fluorescence and excited-state lifetimes. Thus the involvement of LHCBM1 in non-photochemical quenching is suggested to be due to specific interactions with other components of the membrane and not to the inherent quenching properties of the complex. Similarly, the overexpression of LHCBM9 during sulfur deprivation can be explained by its low sulfur content as compared with the other LHCBMs. Considering the highly conserved biochemical and spectroscopic properties, the major difference between the complexes may be in their capacity to interact with other components of the thylakoid membrane.

## Introduction

Photosynthetic light harvesting and electron transfer involve three complexes embedded in the thylakoids membrane: photosystem II (PSII), cytochrome *b*_*6*_ *f* complex (Cyt*b*_*6*_ *f*) and photosystem I (PSI). These complexes drive a linear electron flow (LEF) from water to NADPH that is coupled to the transfer of protons from the stromal to the lumenal side of the membrane, creating a proton gradient that is used by the ATP synthase to produce ATP.

In plants and green algae PSII and PSI are composed of the core complex, which contains the co-factors of the electron transport chain, and the light-harvesting complexes (LHC). The LHCs absorb light and transfer the excitation energy to the reaction centers in the core, where charge separation occurs [1]. In plants, the major LHCII, which acts as an antenna of both photosystems [2], is organized in trimers [3]. Each monomer is composed of three transmembrane helices and coordinates eight chlorophylls (Chl) *a*, six Chls *b* and four xanthophyll molecules (in average 2.4 luteins, 0.6 violaxanthins and one neoxanthin) [4]. Vascular plants contain three major light-harvesting proteins (type 1–3), which in *Arabidopsis thaliana* are encoded by five, four and one genes respectively (*Lhcb1*.*1–1*.*5; Lhcb2*.*1–2*.*4; Lhcb3*.*1*)[5].

Seasonal and diurnal changes, clouds and wind make the photosynthetic organisms constantly exposed to environmental changes. Plants and algae have evolved several strategies to optimize the photosynthetic machinery under different conditions [6] and most of them involve the LHCs. In high light the excess absorbed energy is dissipated as heat through a process called non-photochemical quenching (NPQ) at the level of the LHCs [7,8]. This process limits the formation of reactive oxygen species (ROS) that can damage the photosynthetic apparatus [9,10]. Photosynthetic organisms are also able to modulate the amount of light absorbed by PSII and PSI to optimize the linear electron flow. In the short term, a process called state transitions regulates the association of LHCII to the two photosystems via phosphorylation [6,11]. Transcriptional and translational gene regulation, instead, belong to the long term acclimation responses and regulate the amount of outer antenna in plants, in particular Lhcb1 and Lhcb2, depending on growth conditions [12,13]. In summary, the LHCs have different functions depending on the environmental conditions. They can act as antennas, harvesting light and transferring excitation energy to the RCs or as quenchers, dissipating the energy absorbed in excess to avoid photodamage.

The completely sequenced genome, the vast collection of mutants, easy maintenance and simple life cycle make the unicellular green alga *Chlamydomonas reinhardtii* a popular model system to study biological processes, and especially photosynthesis [14,15] and H2 production [16]. The photosynthetic apparatus of *C*. *reinhardtii* is similar to that of plants and the core complexes of PSI and PSII are highly conserved. The outer antenna is also composed of LHC proteins, but their number and organization differ compared to plants [17–20]. In contrast to *A*. *thaliana*, *C*. *reinhardtii* has nine genes (*LHCBM1–9*) encoding for the major LHCII components. They are divided in four groups based on their sequence homology: Type I (LHCBM3, LHCBM4, LHCBM6, LHCBM8, LHCBM9), Type II (LHCBM5), Type III (LHCBM2, LHCBM7) and Type IV (LHCBM1)[20]. In this manuscript we use the LHCBM nomemclature every time it is possible to directly identify the gene product present in the fractions we analyze, while in the other cases we only indicate the type.

Several studies have suggested a different role for each complex. It was shown that the expression of LHCBM9 increases in response to sulfur deprivation and, more recently, it was suggested that LHCBM9 acts as a quencher in those conditions [21,22]. The absence of LHCBM1 caused a decrease of thermal dissipation (NPQ) but did not affect state transitions [23]; in contrast, LHCBM2/7,LHCBM5 and LHCBM6 were suggested to be involved in state transitions [24–26], although recent results have shown that all LHCBM types can be associated with PSI in state 2 [27]. LHCBM1, -M2 and -M3 are the most abundant LHC in *C*. *reinhardtii* and were found associated with the PSII supercomplexes [19]. LHCBM5 was not observed in the PSII supercomplexes indicating that it is part of the “extra” LHCII population, which is not physically connected to the core [19].

In summary, the data suggest different roles for the individual LHCBMs implying that the complexes have different properties. However, little information is available regarding the individual gene products. In this work we have purified from the membrane the most abundant LHCII subunits in their native state, and we have reconstituted the LHCBMs *in vitro* using the pigments of C. *reinhardtii* and the apoproteins overexpressed in *E*. *coli*. A comprehensive biochemical and spectroscopic characterization of the LHCBMs is presented.

## Materials and Method

### Gene sequences

The sequence data is based on the *C*. *reinhardtii* genome sequence v5.3.1 (Phytozome 10). Chloroplast transit peptides were identified using ChloroP prediction software (http://www.cbs.dtu.dk/services/ChloroP/). The accession number of the genes based on Phytozome nomenclature (NCBI-GenBank accession numbers indicated in parentheses) is: LHCBM1, Cre01.g066917 (AY121229.1); LHCBM2, Cre12.g548400 (XP_001693987.1); LHCBM3, Cre04.g232104 (XP_001703699.1); LHCBM4, Cre06.g283950 (XP_001695344.1); LHCBM5, Cre03.g156900 (XP_001697526.1); LHCBM6, Cre06.g285250 (XP_001695353.1); LHCBM7, Cre12.g548950 (XP_001694115.1); LHCBM8, Cre06.g284250 (XP_001695467.1); and LHCBM9, Cre06.g284200 (XP_001695466.1).

### Strain, growth conditions and thylakoids preparations

The growth of *C*. *reinhardtii* (strain JVD-1B[pGG1]) cells and the isolation of the thylakoid membrane were performed as described in [28] with the modification described in [18]. Briefly, the cells were grown in liquid Tris-Acetate-Phosphate medium (TAP) at room temperature (25°C) shaking at 170 rpm in 50 μmol photons PAR m^-2^ s^-1^. For thylakoid preparation, the cells were disrupted by sonication (60W power in 10 cycles of 10s on/30 s off) and centrifuged at 15000 rpm at 4°C for 20 min. Purification of thylakoid membrane was made using a discontinuous gradient in a SW41 swinging bucket rotor (24000 rpm, 1 h, 4°C).

### Isolation of PSII light harvesting complexes

Thylakoids were pelleted, unstacked with 5mM EDTA and washed with 10mM HEPES (pH 7.5). Membranes were then resuspended in 20mM Hepes (pH 7.5), 0.15M NaCl and solubilized at the final Chl concentration of 0.5 mg/ml by adding an equal volume of 0.6% α-dodecylmaltoside (α-DM). Unsolubilized material was eliminated by centrifugation (12000 rpm for 10 min at 4°C). Solubilized thylakoids were loaded on a sucrose density gradient made by freezing and thawing 0.65 M sucrose, 10mM Tricine (pH 7.8), 0.03% α-DM buffer, and separated by ultracentrifugation in a SW41 rotor at 41000 rpm for 14 hours at 4°C. The green bands were harvested with a syringe.

### Non-denaturing Isoelectrofocusing (ndIEF)

The gradients bands containing monomeric and trimeric Lhcs were subjected to non-denaturing flat-bed iso-electro-focusing (ndIEF) as described in [29]. Briefly, a bed (100 ml) of 5% ultrodex (LKB), 2% ampholites carrier (pH 3.5–5), 1% glycine and 0.06% β-DM was prepared and dried to form a 4 mm gel layer. Samples, mixed with 2% of ampholites were loaded on the gel and focused with a constant power of 9 W for 15 hours at 4°C. The green bands were harvested with a spatula and the sample eluted from the gel using a solution containing 50 mM HEPES pH 7.6 and 0.03% α-DM. The fraction were loaded on sucrose density gradients made by freezing and thawing 0.5 M sucrose, 0.06% β-DM and 20 mM Hepes (pH 7.5) and centrifuged at 41000 rpm for 14 hours at 4°C using a SW60 swinging bucket rotor. The green bands were harvested with a syringe.

### DNA Cloning and Recombinant protein overexpression

*LhcbM1*, *-M2*, *-M5*, *-M6 and -M9* genes of *C*. *reinhardtii* were amplified from a cDNA library (Chlamydomonas Resource Center Database) by PCR and cloned into a pET-His expression vector. Primers were designed to remove the stop codon and create recombinant proteins carrying 6 his residues at the C-terminal (S1 Table). Heat shock transformation was used to transform *E*. *coli* DH5-α cells (New England Biolabs) with the recombinant vectors. His-tagged apoproteins were overexpressed by growing the bacteria with Isopropyl β-D-1-thiogalactopyranoside (IPTG) at 37°C overnight. Finally, inclusion bodies were purified as described in [30] with the modifications reported in [31].

### *In vitro* reconstitution and purification of refolded LHCII

These procedures were performed as described previously [31]. Briefly, the apoprotein was denatured by heat in the presence of lithiumdodecylsulfate (LDS), followed by the addition of pigments (extracted from *C*. *reinhardtii*) with a Chl *a*/*b* ratio of 2.5 and Octyl β-D-glucopyranoside (OG). LDS was then removed by precipitation upon addition of KCl. The refolded LHC was purified from free pigments and unspecific products by Ni-affinity chromatography and sucrose gradient centrifugation. We performed three biological replicas per each sample. The same procedure was applied to reconstitute LHCBM1 using pigments extracted from spinach leaves.

### SDS-PAGE

Proteins were analyzed by SDS-6M urea PAGE with Tris-Tricine buffer system as in [32] using 14% acrylamide concentration in the running gel. The Coomassie stained gels were imaged with ImageQuant LAS-4000 (GE Healthcare).

### Pigment extraction and HPLC analysis

The pigment composition of the complexes was analysed by fitting the spectrum of the pigments extracted in 80% acetone with the spectra of the individual pigments in the same solvent and by HPLC, as described previously [33]. The reported values are the average of three repetitions.

### Absorption, Circular Dichroism and steady state fluorescence measurements

Room temperature absorption spectra were recorded with a Cary 4000 spectrophotometer (Varian). The CD spectra were measured using a Chirascan CD Spectrophotometer (AppliedPhotophysics). The fluorescence emission spectra were recorded using a Fluorolog 3.22 spectrofluorimeter (Jobin Yvon-Spex). The excitation wavelengths were 440nm, 475nm and 500nm and emission was detected in the 600–800nm range. Excitation and emission bandwidths were set to 3 nm. All fluorescence spectra were measured at OD 0.05 at the maximum of the Qy absorption band. Room temperature measurements were performed in 0.5M sucrose, 20mM Hepes (pH 7.5), 0.06% β-DM buffer. For 77K measurements a liquid nitrogen cooled device was used (Nitrogen Cryostat, Oxford Instruments). The samples were in 70% glycerol (w/v), 20 mM Hepes (pH 7.5), 0.06% β-DM buffer.

### Time-correlated single photon counting (TCSPC)

Time-resolved fluorescence measurements were performed using a FluoTime 200 fluorometer (PicoQuant). The samples were diluted to an OD of 0.05 cm^−1^ at the Qy maximum, stirred in a cuvette with a path length of 1 cm. Concerning the pH-dependent experiments on LHCBM1, the pH exchange was performed as reported previously [34] by loading the reconstituted protein on a sucrose gradient containing 20 mM MES pH 5.5. Excitation was provided at 10 MHz repetition rate by a 470nm laser diode. The instrument response function was obtained with pinacyanol iodide in methanol, which has 6-ps fluorescence lifetime [35]. Fluorescence emission was collected at 90° with respect to the excitation, at 680 nm. All measurements were performed at 283K, and the maximum number of counts in the peak channel was 20000.

## Results

**Fig 1:**
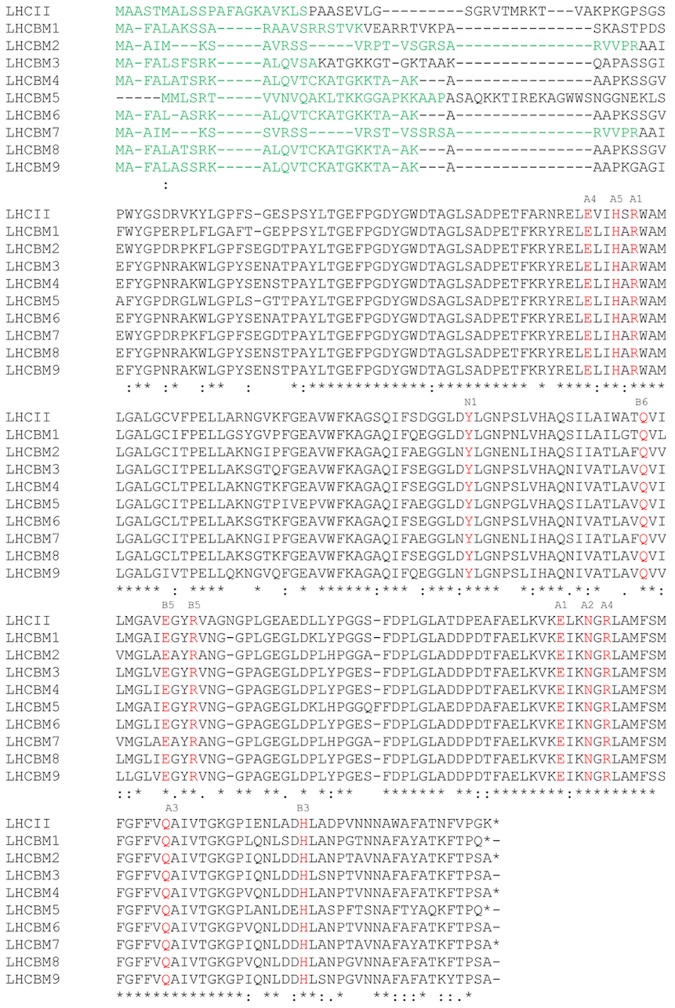
*In silico* analysis of LHCBM proteins. Multiple sequence alignment of *C*. *reinhardtii* LHCBM proteins (Clustal OMEGA). Red-colored amino acids indicate the conserved Chl a and Chl b binding sites and the neoxanthin binding site (tyrosine, N1) [36]; green colored amino acids indicate the transit peptide; blue colored amino acids indicate the region of the trimerization motif.

The LHCII proteins forming the major antenna system of *Chlamydomonas reinhardtii* (*LHCBM1–9*) show high homology (between 75 and 80%) (Fig. 1). It should be noted that LHCBM2 and M7 encode for the same mature protein, which makes it impossible to know which of the two is present in our purified fractions. In the following we will then indicate this complex as LHCBM2/7. The nine LHCBMs also show high homology with the LHCII of plants: All chlorophyll ligands observed in the structure of plant LHCII [4] as well as the tyrosine that is essential for the binding of neoxanthin [36], are conserved in the 9 LHCBMs. The WYxxxR sequence at the N-terminus which is necessary [37] for the trimerization of LHCII in plants, is fully conserved in LHCBM1 and LHCBM2/7, while W is replaced by F in the other LHCBMs.

The theoretical pI values of the individual LHCBMs were calculated based on the mature protein sequence of each complex (http://web.expasy.org/compute_pi/). The pI values were found to range between 4.71 and 5.42 with LHCBM5 exhibiting the highest value (Table 1). Atomic composition analysis shows that LHCBM9 contains only 4 sulfur atoms whereas this number is 6 or 7 for the other LHCBMs (Table 1).

**Table 1:** Isoeletric point and number of sulfur atoms of the LHCBM proteins.

	pI	N° of sulfur atoms
**LHCBM1**	**4.99**	**7**
**LHCBM2**	**4.71**	**7**
**LHCBM3**	**5.27**	**6**
**LHCBM4**	**4.88**	**7**
**LHCBM5**	**5.42**	**7**
**LHCBM6**	**4.88**	**7**
**LHCBM7**	**4.71**	**7**
**LHCBM8**	**4.88**	**7**
**LHCBM9**	**4.87**	**4**
**LHCII**	**5.17**	**7**

LHCII refers to *Arabidopsis thaliana* antenna complex LHCB2.

### Purification and characterization of the native complexes

**Fig 2:**
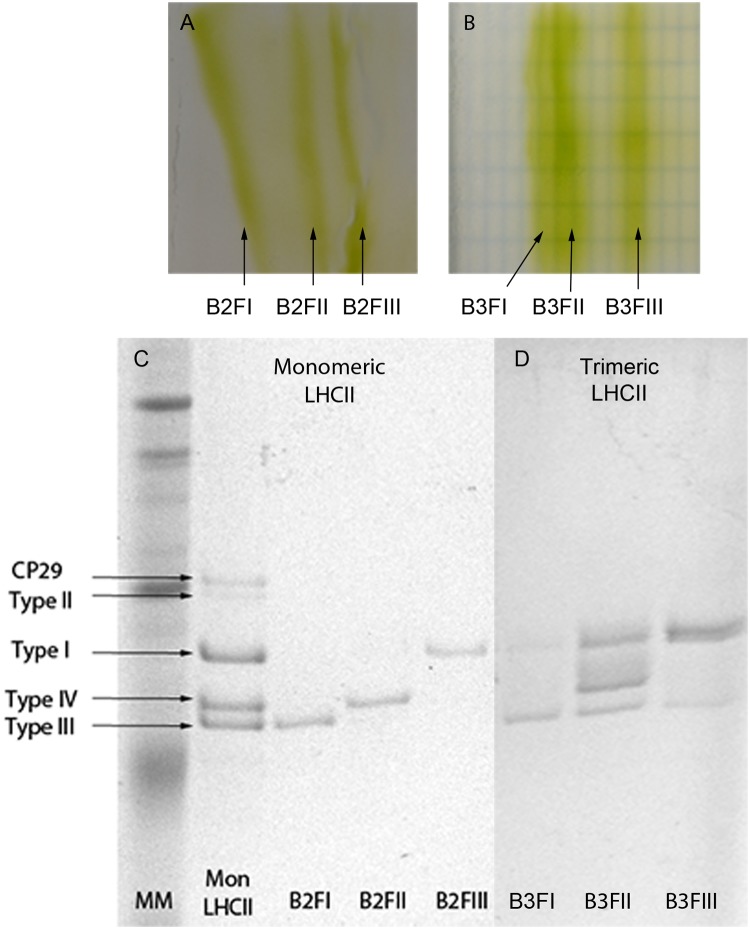
Tricine SDS-PAGE of the ndIEF fractions. (A) Monomeric LHCII and (B) Trimeric LHCII separated by ndIEF. (C) Monomeric LHCII and (D) Trimeric LHCII ndIEF fractions separated by SDS-PAGE. MM: Molecular Marker; Mon LHCII: Monomeric LHCII isolated from a sucrose gradient purification. B2FI, B2FII, B2FIII: Different fractions from the LHCII monomer. B3FI, B3FII, B3III: Different fractions from the LHCII trimers.

In order to study the properties of the individual LHCBM subunits, the thylakoid membranes were solubilized and fractionated on sucrose gradients [19]. Bands 2 and 3 of the gradient contained a combination of different LHCBMs in their monomeric and trimeric states, respectively (data not shown). To purify the individual complexes we took advantage of the expected differences in their pIs and subjected the bands harvested from the gradient to non-denaturing iso-electrofocusing (ndIEF). For both monomeric and trimeric complexes, three well- separated green fractions with similar intensity were obtained (Fig. 2A-B) which run as single bands when loaded onto sucrose gradients. Neither pellet nor free pigments were present in the sucrose gradient. The protein composition of each band was analyzed by SDS-PAGE (Fig. 2C) and the identity of each band attributed on the bases of previous work in which the individual bands were analyzed by mass spectrometry [19,26,38]. The most acidic monomeric fraction (B2FI) contained LHCBM2/7 (type III), the second (B2FII) LHCBM1 (type IV) and the third one (B2FIII) some of the type I LHCBMs (M3/4/6/8/9) (Fig. 2C). In the case of the trimers, the most acidic fraction (B3FI) contained mainly LHCBM2/7 and some traces of type I LHCBMs, the second (B3FII) type I, LHCBM2/7 and LHCBM1 in 0.7/1/0.2 ratio (calculated on the basis of the stain), and the third (B3FIII) type I and a small amount of LHCBM2/7 (Fig. 2D). We were not able to detect LHCBM5 in the monomeric or trimeric form, probably due to the low amount of this complex in the membrane.

**Fig 3:**
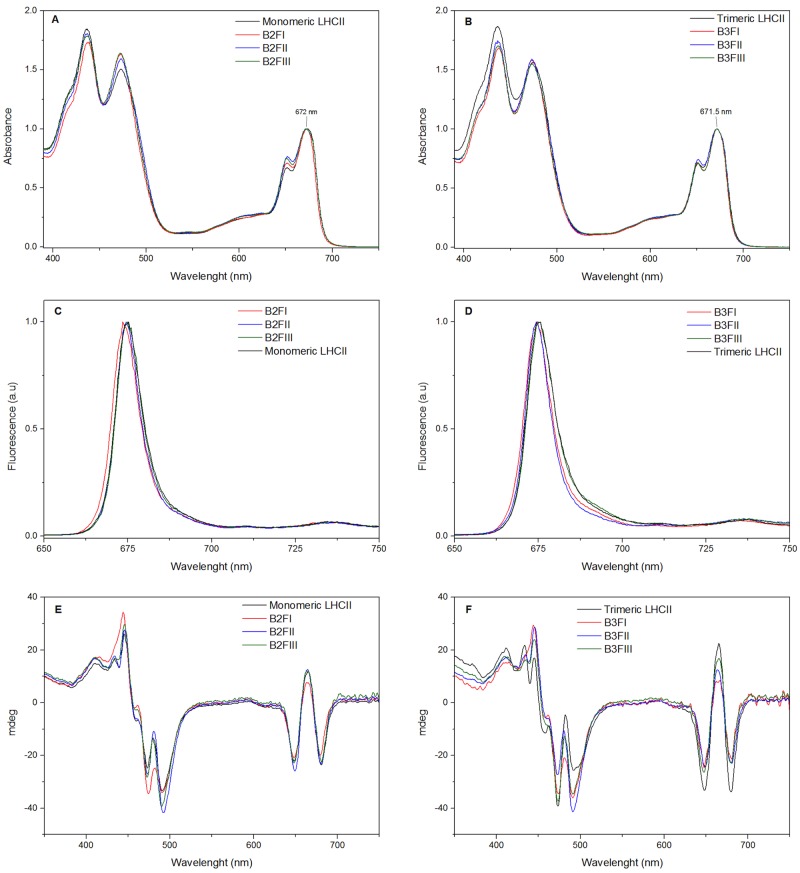
Spectroscopic analysis of the ndIEF fractions. (A) and (B): Absorption spectra of ndIEF fractions. (C) and (D): Fluorescence emission spectra at 77K (excitation at 440 nm). (E) and (F): Circular dichroism spectra at 10°C. B2FI, B2FII, B2FIII: Different fractions from the LHCII monomer. B3FI, B3FII, B3III: Different fractions from the LHCII trimers.

The absorption spectra of all fractions are reported in Fig. 3. The maximum in the Q_y_ region is at 671.5–672nm for both monomers (Fig. 3A) and trimers (Fig. 3B), thus 2 nm blue-shifted as compared to LHCII of higher plants. A second peak at 651 nm, typical of LHCII [39] and representing mainly Chl *b* absorption, was observed in all samples (Fig. 3A-B).

The fluorescence emission spectra at low temperature (77K) of monomeric and trimeric complexes exhibited a maximum around 677 nm. Small differences in the spectra could be observed between the individual LHCBMs: The spectrum of both monomeric and trimeric LHCBM2/7 (B2FI and B3FI) was 1–1.5 nm blue-shifted, while that of B3FIII (Fig. 3C-D) was slightly red-shifted and showed increased intensity above 690 nm.

Circular dichroism (CD) in the visible region is sensitive to the coupling between pigments [40] and can thus be used to determine differences in the pigment organization between complexes. The spectra of the monomeric and trimeric LHCIIs are presented in Fig. 3E and 3F, respectively. In the Qy absorption region all spectra showed the typical-+- signal of LHCII. The relative intensities of the bands were also similar, indicating a conserved Chl organization. Small differences were visible in the blue region dominated by Chl/carotenoid interactions [41]. Remarkably the spectra of monomer and trimers are more similar than observed for the complexes of higher plants, [41] suggesting that the trimerization does not have a large effect on the carotenoid organization/spectral properties in *C*. *reinhardtii*.

The pigment composition of the purified fractions was analyzed by HPLC and fitting of the acetonic extract (Table 2). The Chl *a/b* ratio of all fractions was lower (around 1.1) as compared to that of the starting LHCII preparations (1.30). The amount of violaxanthin was also lower after IEF (0.1–0.2 vio/monomer vs 0.5 vio/monomer) (Table 2). These differences indicate loss of pigments, in particular Chl *a* and violaxanthin, during IEF. Similar results were obtained for LHCII from barley [42].

**Table 2:** Pigment composition of the LHCII fractions generated by ndIEF.

Sample	Chl *a*/chl *b*	Chls/car	Neo+Loro/mon	Vio/mon	Lut/mon	Chl *b*/mon	Chl *a*/mon	Cars/mon	Chl
**LHCIImonomer**	1.30±0.05	3.77±0.05	1.86±0.02	0.55±0.01	1.28±0.01	6.06±0.14	7.93±0.14	3.71±0.05	14
**B2FI**	1.03±0.01	4.73±0.07	1.75±0.03	0.15±0.01	0.84±0.01	6.4±0.01	6.6±0.01	2.74±0.04	13
**B2FII**	1.16±0.02	4.33±0.08	1.90±0.02	0.14±0.01	0.94±0.03	6.01±0.06	6.98±0.06	2.99±0.05	13
**B2FIII**	1.10±0.01	4.32±0.04	1.88±0.02	0.18±0.01	0.94±0.02	6.16±0.02	6.83±0.02	3.00±0.02	13
**B3FI**	1.14±0.04	4.07±0.13	1.82±0.08	0.21±0.06	1.15±0.02	6.06±0.13	6.93±0.13	3.19±0.10	13
**B3FII**	1.14±0.03	4.61±0.07	1.82±0.04	0.12±0.01	0.87±0.01	6.05±0.09	6.94±0.09	2.81±0.04	13
**B3FIII**	1.14±0.01	4.51±0.12	1.79±0.07	0.18±0.01	0.90±0.01	6.07±0.01	6.92±0.01	2.88±0.07	13

The data are the results of three pigment extraction replicas.

**Fig 4:**
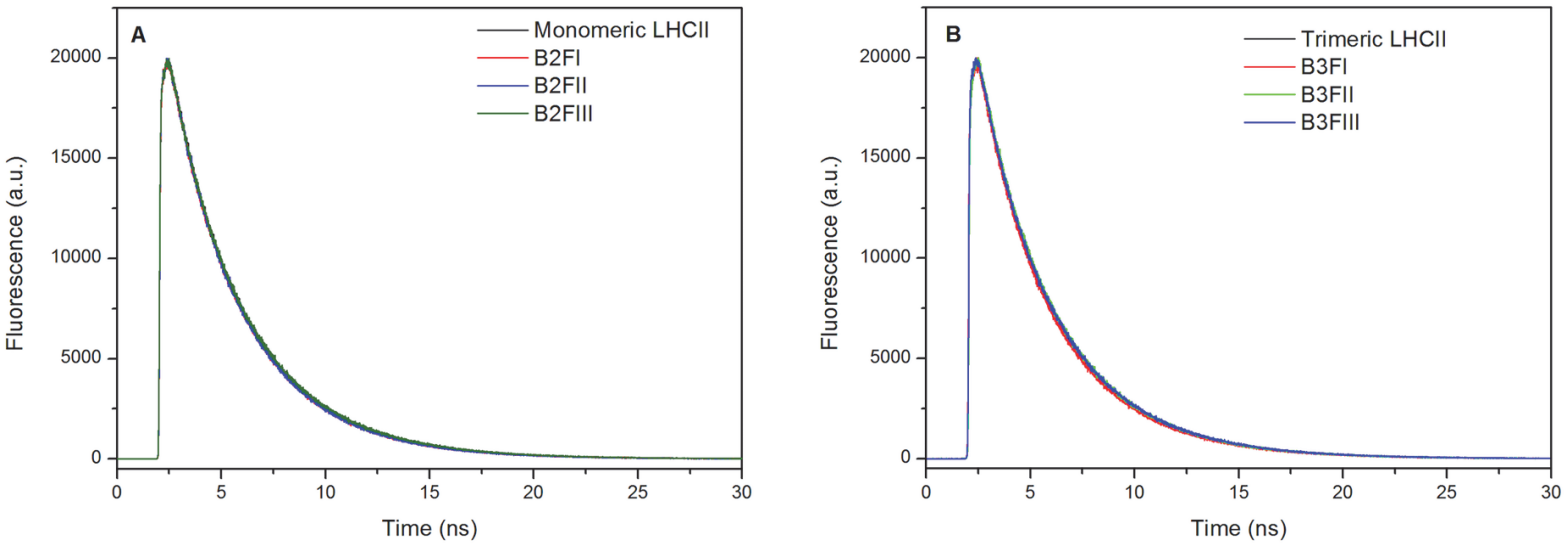
Time-Resolved Fluorescence Decay kinetics of the ndIEF fractions. (A) monomeric complexes; (B) trimeric complexes.

To determine if the proposed role in quenching of some of the complexes was due to their intrinsic properties, as previously suggested [22], the excited state lifetimes of the complexes were measured by time-resolved fluorescence. After excitation at 470 nm we recorded the emission at 680 nm for all fractions (Fig. 4A-B). The fluorescence decay kinetics could be satisfactorily described with three components (Table 3): a fast component with lifetime around 0.5 ns, which was present in all samples and accounted for ~10% of the amplitudes; a second component of ~3.2 ns with the largest amplitude; and a longer component of 4–5 ns. The average lifetime was around 3.2–3.3 ns for all fractions, remaining practically identical to the value observed before ndIEF. This indicates that violaxanthin in the V1 site does not influence the excited state lifetimes of the complexes which remains compatible with the light harvesting conformation.

**Table 3:** Excited state Lifetimes of the LHCBM monomeric and trimeric fractions.

Protein	τ_ave_, ns	A_1_, %	τ_1_, ns	A_2_, %	τ_2_, ns	A_3_, %	τ_3_, ns
**B2FI**	3.26	11	0.57	73	3.27	17	4.91
**B2FII**	3.23	10	0.50	63	3.12	26	4.57
**B2FIII**	3.22	12	0.31	41	2.82	47	4.29
**B3FI**	3.25	11	0.51	61	3.12	29	4.53
**B3FII**	3.39	9	0.57	81	3.44	10	5.37
**B3FIII**	3.32	12	0.53	73	3.39	15	5.17
**B3 (trimeric LHCII)**	3.30	10	0.53	64	3.17	26	4.67
**B2 (monomeric LHCII)**	3.27	10	0.35	49	2.92	41	4.37

The amplitudes (A_i_) of the different lifetimes (t_i_) to the fluorescence decay components, and the average fluorescence lifetime (t_ave_) of the samples are given.

### *In vitro* reconstitution of the individual LHCBMs

Although several fractions containing different LHCBMs could be separated by ndIEF, due to the high sequence homology and the low abundance of most complexes only LHCBM1 and LHCBM2/7 could be purified to homogeneity. To study the individual complexes, we have then reconstituted them *in vitro* using the pigments extracted from *C*. *reinhardtii* and the apoprotein overexpressed in *E*. *coli*.

We reconstituted five complexes: LHCBM1 and LHCBM2, which can be directly compared with the native complexes purified by ndIEF; and LHCBM5, LHCBM6 and LHCBM9, which were suggested to have different roles and different properties [19,22,23,26]. The quality of the reconstitution was assessed by measuring the fluorescence emission spectra of the LHCBMs upon preferential excitation of Chl *a*, Chl *b* and carotenoids. The shape of all spectra was identical, demonstrating efficient excitation energy transfer within the complexes (S1 Fig.).

**Fig 5:**
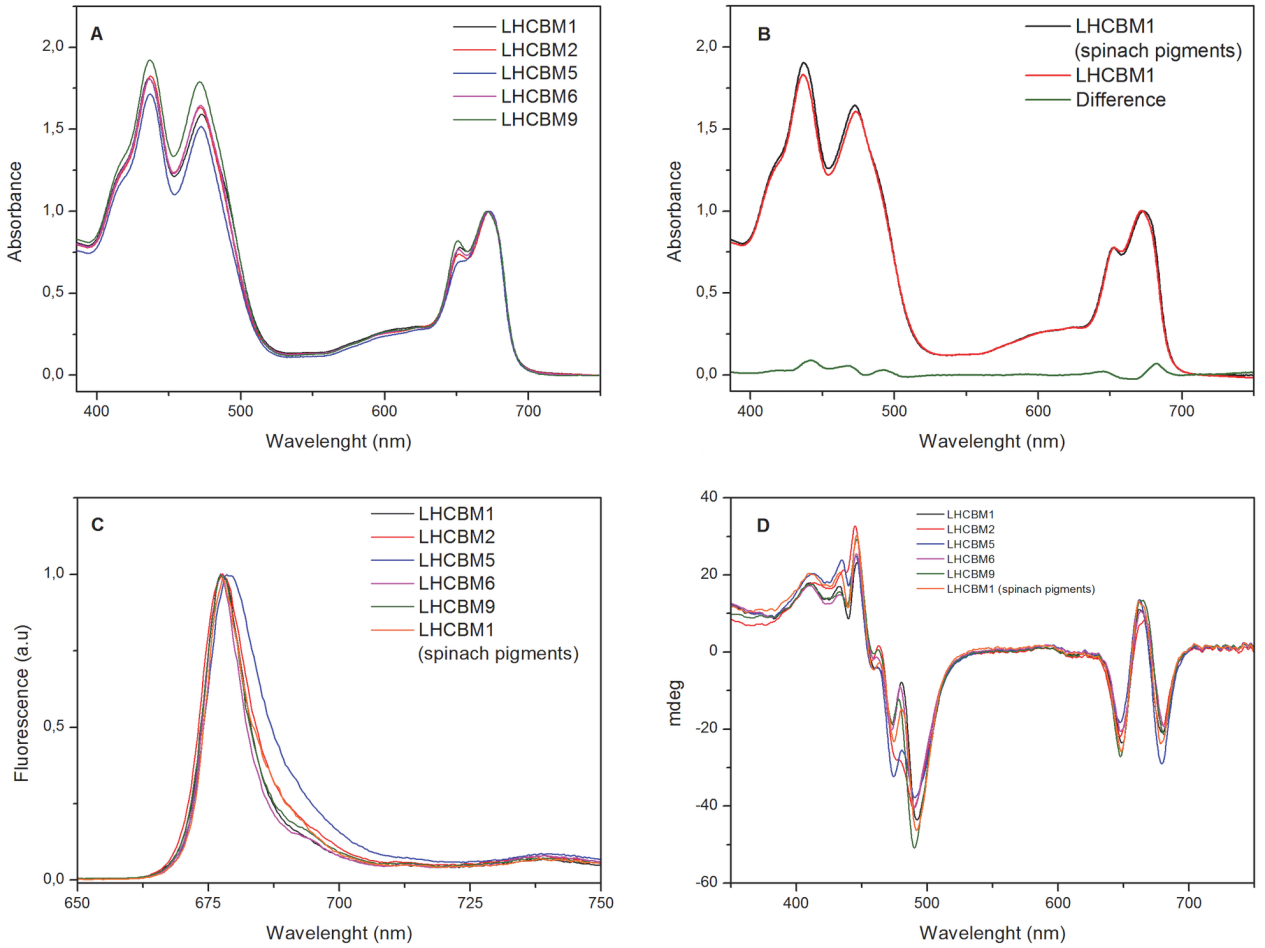
Absorption spectra (A)–(B), Fluorescence emission spectra at 77K (excitation at 440 nm) (C) and Circular Dichroism (D) of the LHCBM reconstituted complexes. The absorptions are normalized to the Q_y_ peaks. Fluorescence emission spectra are normalized to the maximum, while the CD signals are normalized on the absorption spectra.

The absorption spectra of the reconstituted complexes are reported in Fig. 5A. In general, all complexes show similar features, with the Qy peaks at 671.5 and 651 nm and the Soret transitions at 439 nm and 466 nm as for the isolated complexes. The presence of small differences in the spectra could indicate changes in the pigment content, and/or in pigment-protein and pigment-pigment interactions. LHCBM5 showed lower intensity at 650 nm as compared to the other LHCBMs.

The Chl *a/b* ratio of the individual LHCBMs ranges from 1.08 of LHCBM9 to 1.26 of LHCBM5 (Table 4). The differences in the pigment composition between the complexes were observed in three biological replicas, although the absolute values changed slightly. It should be mentioned that the reconstitutions of all complexes were done simultaneously using the same pigment mix in order to directly compare the properties of the complexes. All LHCBMs contain lutein, loroxanthin and neoxanthin and a small amount of violaxanthin. To determine in which site loroxanthin binds, we reconstituted LHCBM1 with the pigments isolated from spinach, which do not contain loroxanthin. The Chl *a/b* ratio and the Chl/car ratio were very similar to those of the complex reconstituted with *C*. *reinhardtii* pigments, but in the LHCBM1-spinach the amount of lutein was strongly increased, indicating that loroxanthin is substituting lutein in one of the two internal binding sites, as suggested previously [19]. Interestingly, the absorption spectrum of LHCBM1-spinach shows the maximum in the Qy at 673.5 nm in line with that of the plants LHCII (Fig. 5B).

**Table 4:** Pigment composition of the LHCBM reconstituted proteins *in vitro*.

Sample	Chl *a*/ chl *b*	Chls/car	Neo+loro/Mon	Vio/mon	Lut/mon	Chl *b*/mon	Chl *a*/mon	Cars/mon	Chl
**LHCBM1**	1.18±0.01	4.82±0.04	1.45±0.03	0.11±0.01	1.32±0.01	6.42±0.01	7.57±0.01	2.90±0.02	14
**LHCBM2**	1.20±0.01	5.07±0.04	1.22±0.02	0.10±0.02	1.43±0.04	6.35±0.04	7.64±0.04	2.76±0.02	14
**LHCBM5**	1.26±0.01	5.40±0.07	1.11±0.01	0.09±0.02	1.38±0.01	6.18±0.04	7.81±0.04	2.59±0.03	14
**LHCBM6**	1.14±0.01	4.93±0.07	1.47±0.02	0.10±0.01	1.25±0.01	6.52±0.01	7.47±0.01	2.83±0.04	14
**LHCBM9**	1.08±0.01	4.60±0.25	1.61±0.01	0.24±0.01	1.19±0.06	6.70±0.03	7.29±0.03	3.04±0.16	14
**LHCBM1(spinach pigments)**	1.22±0.02	4.71±0.1	0.84±0.02	0.13±0.01	1.99±0.07	6.29±0.06	7.70±0.06	2.97±0.10	14

The values are normalized to 14 chls (*a+b*). The data are the results of three preparations in three replicas.

The 77K fluorescence emission spectra of most reconstituted complexes are very similar, peaking around 677 nm and showing a similar FWHM (Fig. 5C). Only the spectrum of LHCBM5 is slightly red shifted (max 679 nm) and broader, with increased emission in the red region as compared to the other complexes (Fig. 5C). The spectrum of LHCBM1-spinach is 1 nm red-shifted and shows a smaller FWHM compared to LHCBM1-Chlamy.

As observed for the native complexes, the CD spectra of the reconstituted proteins show the typical LHCII features (Fig. 5D). LHCBM1, -M2, -M6 and -M9 are almost identical in the Qy absorption region and LHCBM1, -M6 and -M9 are also almost identical in the blue region, indicating a very similar pigment organization. LHCBM2 differs from the other complexes in the intensity of the band at 480 nm. The spectrum of LHCBM5 shows a different ratio between the 650 nm and 680 nm, and the 475 nm and 490 nm components, which probably reflects its lower Chl *b* content (Fig. 5D).

**Fig 6:**
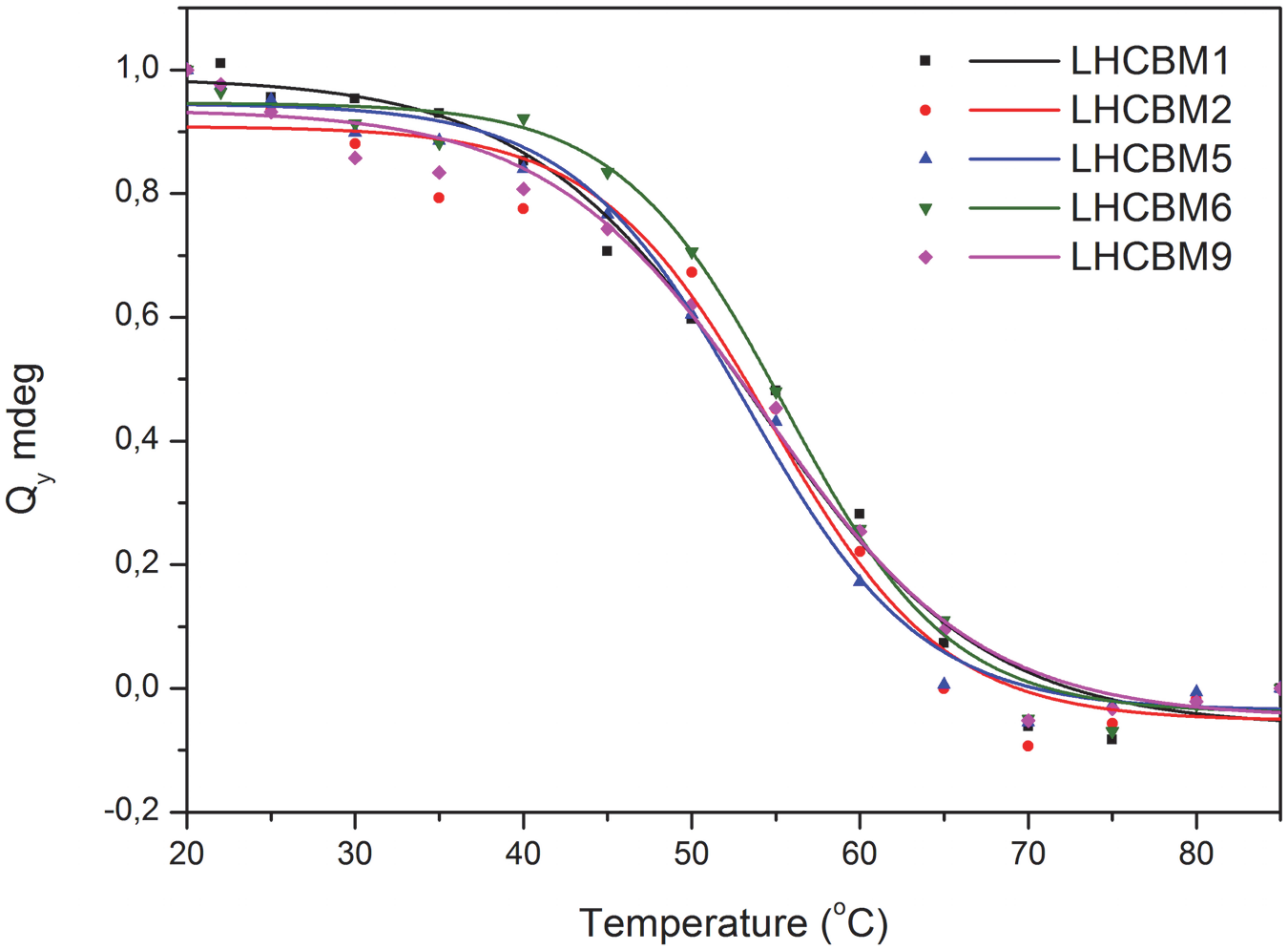
Changes in the CD signal of the LHCBM reconstituted proteins at 680 nm as a function of temperature. Black line represents the fitting of the denaturation.

The stability of the complexes was tested by measuring the temperature denaturation curve as obtained by monitoring changes in the CD spectra between 630 nm and 700 nm while increasing the temperature from 20°C to 90°C (S2 Fig.). As shown in Fig. 6 all complexes show a denaturation temperature of ~55°C.

The fluorescence decay kinetics of the reconstituted complexes were measured in the same conditions as described above for the isolated fractions. The average lifetime for most of the complexes was around 2.7–2.9 ns, the only exception being LHCBM5 that showed an average lifetime of 2.4 ns (Table 5). The lifetimes of LHCBM1 reconstituted with either lutein or loroxanthin were identical.

**Table 5:** Decay kinetics of the different LHCBM reconstituted proteins.

Protein	τ_ave_, ns	A_1_, %	τ_1_, ns	A_2_, %	τ_2_, ns	A_3_, %	τ_3_, ns
**LHCBM1 (spinach pigments)**	2.67	15	0.49	54	2.49	31	4.08
**LHCBM1**	2.87	15	0.65	70	2.93	15	4.85
**LHCBM2**	2.78	19	0.73	60	2.80	21	4.54
**LHCBM5**	**2.43**	24	0.62	51	2.35	26	4.25
**LHCBM6**	2.93	13	0.37	45	2.53	42	4.17
**LHCBM9**	2.7	16	0.56	58	2.58	27	4.25

The amplitudes (A_i_) of the different lifetimes (t_i_) to the fluorescence decay, and the average fluorescence lifetime (t_ave_) are given.

## Discussion

**Fig 7:**
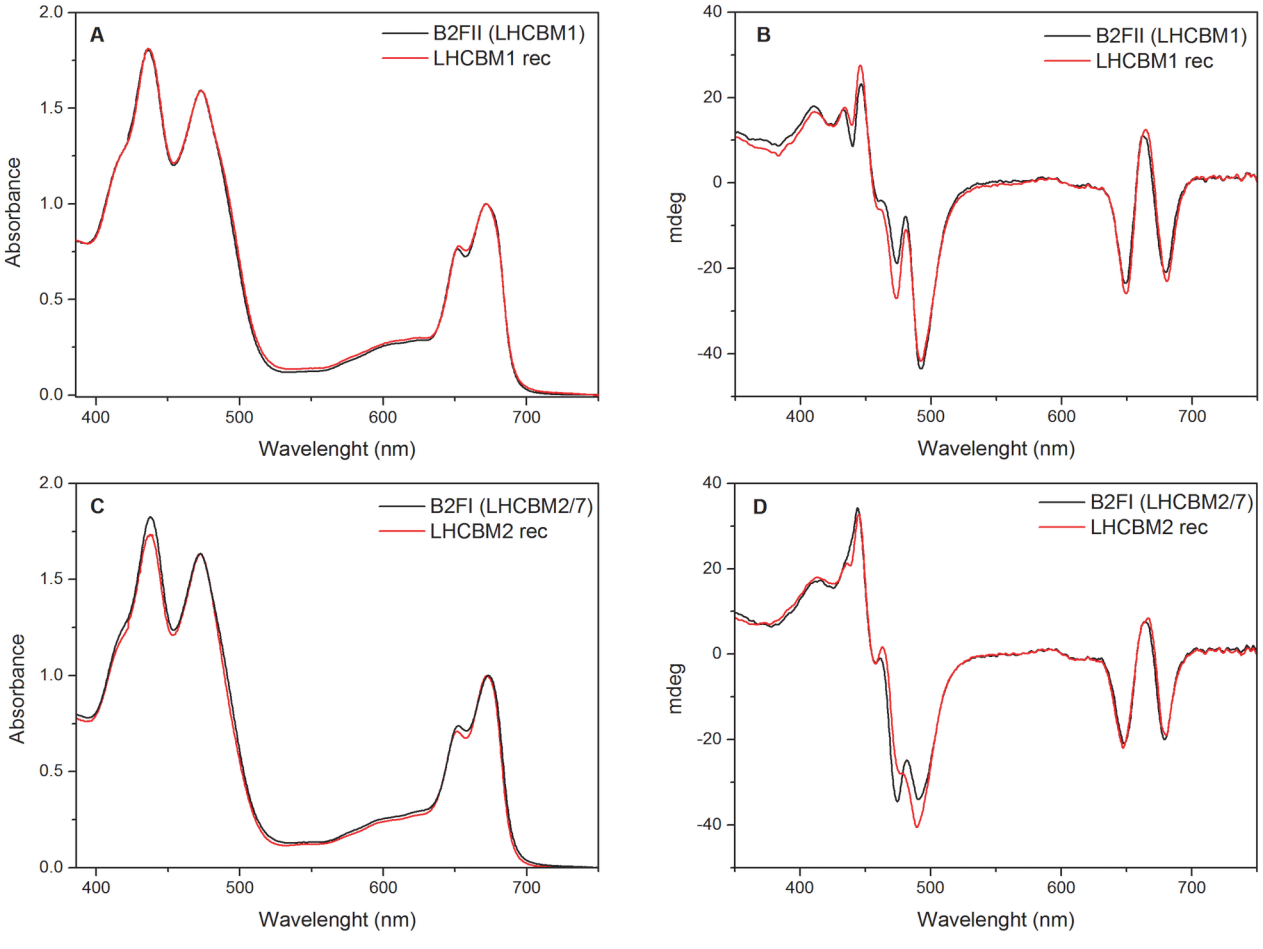
Comparison of the absorption (A–C) and Circular Dichroism (B–D)) of LHCBM1, LHCBM2 reconstituted and the purified fractions from ndIEF containing LHCBM1 and LHCBM2/7. The absorptions are normalized to the Q_y_ peaks, while the CD signals are normalized on the absorption spectra.

To study the properties of the LHCBMs of *C*. *reinhardtii*, we have taken advantage of the differences in pI between the LHCBMs to purify them from solubilized membranes. This procedure has allowed us to obtain homogeneous preparations of monomeric LHCBM1, and monomeric and trimeric LHCBM2/7. However, the high sequence homology and the low amount of the other LHCBM complexes prevented us from purifying them from the membranes. To overcome this problem we reconstituted them *in vitro* [43], a procedure that in plants was shown to lead to complexes identical to the native ones [44,45]. The comparison of the properties of LHCBM1 and LHCBM2/7 (the mature proteins of -M2 and -M7 have identical sequence) purified from the membrane, with those of the same complexes reconstituted *in vitro* allow us to check the validity of the reconstitution procedure also for *C*. *reinhardtii* gene products. The absorption spectra of recombinant and native LHCBM1 are almost identical, with the CD spectrum, which is highly sensitive to pigment organization and coupling, being very similar in the red region and showing only small differences in the Soret region (Fig. 7A-B). The same similarity is visible for LHCBM2/7 (Fig. 7C-D). Only the excited-state lifetimes of the recombinant complexes are 10–15% shorter than those of the native monomers. The multi-exponential fluorescence decay observed for the Lhcs of plants were ascribed to the presence of different conformations with different degrees of quenching [46,47]. It is thus likely that the reconstitution *in vitro* moves the equilibrium slightly towards more quenched conformations. However, in general we can conclude that the recombinant complexes have the same properties as the native ones and can be used for the characterization of the low abundant complexes.

The pigment complement of all LHCBMs is very similar and it is also similar to that of LHCII from plants. The data indicate that ndIEF leads to loss of violaxanthin and lutein, suggesting that they are accommodated in the V1 site, as in plants [42]. Interestingly, upon ndIEF also a loss of Chl *a* was observed, again as in plant LHCII [42]. One possible candidate is Chl 614, which is located at the periphery of the complex. Indeed, the ligand for Chl 614 is a histidine that can be protonated at low pH thus leading to the loss of Chl coordination. After ndIEF, 1 neoxanthin, 1 loroxanthin and 1 lutein are still associated with each complex, with neoxanthin in the N1 site, as suggested by the presence of the Tyr which stabilizes its binding [36] and lutein and loroxanthin in L1 and L2.

The high similarity between the LHCBM of *C*. *reinhardtii* and LHCII of plants suggests that the Chl binding sites are also conserved. However, the absorption spectra of all LHCBMs is 2 nm blue-shifted as compared to that of LHCII of plants, suggesting differences in the lowest energy site of the complexes. Interestingly, this blue shift was not observed when LHCBM1 was reconstituted with the pigments of spinach, which accommodated lutein in both L1 and L2 sites. The difference spectrum between LHCBM1 with lutein and with loroxanthin (Fig. 5B) shows a main positive component at 682 nm, indicating that the presence of loroxanthin affects the lowest energy Chls. In all plants Lhc the lowest energy Chls are associated with Chl 610, 611 and 612, which are located close to the L1 site [48–50]. We therefore speculate that loroxanthin in LHCBMs is located in the L1 site and influences the environment of these Chls.

Different roles in light harvesting and photoprotection have been proposed for the individual LHCBMs which might depend on their intrinsic properties. However, the excited-state lifetimes are very similar for all complexes with the exception of LHCBM5, which has a somewhat shorter lifetime (2.4 ns), accompanied by a red-shifted fluorescence spectrum. On the contrary, we could not see significant differences between LHCBM1 and -M9, which are suggested to be involved in quenching, compared to the other complexes, which should mainly be involved in light harvesting. As the absence of LHCBM1 strongly reduces the NPQ level *in vivo* [23], we have also tested its capacity to respond to pH changes, which *in vivo* represents the trigger for the activation of NPQ [8,34]. However, no differences in the lifetimes of the complex at different pHs were observed (Table 6), confirming what was previously found for the homologous LHCII of higher plants [34]. We thus conclude that the quenching properties of LHCBM1 in the membrane are not the result of a direct influence of pH or due to intrinsic properties of the protein as previously proposed [22], but most probably the result of interactions with other complexes, e.g. LHCSR [51].

**Table 6:** Decay kinetics of LHCBM1 reconstituted *in vitro* at different pH.

pH	τ_ave_, ns	A_1_, %	τ_1_, ns	A_2_, %	τ_2_, ns	A_3_, %	τ_3_, ns
**7.6**	2.73	14	0.49	33	2.01	51	3.78
**5.5**	2.61	17	0.43	35	2.04	48	3.81

The amplitudes (A_i_) of the different lifetimes (t_i_) to the fluorescence decay, and the average fluorescence lifetime (t_ave_) are given.

LHCBM9 was shown to be over-expressed during sulfur deprivation [21] and it was suggested that it quenches PSII supercomplexes [22]. Here we show that LHCBM9 is not quenched in solution, which implies that switching to a quenched state may depend upon its association with the PSII supercomplex. However, the lower sulfur content of LHCBM9 also indicates that the expression of this protein is part of the acclimation response of *C*. *reinhardtii* aimed at preventing the consumption of sulfur [21] and we speculate that this is the main reason for its overexpression during S deprivation. We propose that the two methionines conserved in the mature sequence of LHCBM9 may be essential for its functionality (and possibly stability).

Finally, the purification of the complexes from the membrane indicates that LHCBM1, LHCBM2/7 and type I LHCBM (3/4/6/8/9) are present in comparable amounts in *C*. *reinhardtii*. Due to the very high sequence homology it was not possible to separate the individual type I complexes; however, previous mass spectrometry analysis has shown that the most abundant type I complex is LHCBM3 [19]. In the monomeric state the different LHCBM types could be purified to homogeneity, while for the trimers this was only possible for LHCBM2/7 and type I LHCBMs, indicating that they can form homotrimers. Instead, LHCBM1 was found in a fraction also containing other LHCBMs, suggesting that it forms heterotrimers.

After having thoroughly characterized the individual LHCBMs, the next step is to understand their capacity to interact with other components of the membrane using liposomes or model membrane systems.

## Supporting Information

S1 Fig Fluorescence emission spectra of the different reconstituted proteins at room temperature. The emission was recorded after excitation at 440 nm, 475 nm and 500 nm. The data are normalized to the maximum.
(TIF)

S2 Fig Circular Dichroism Signal during the increasing of temperature of LHCBM2 protein. (JPG)

S1 Table Primer list of the different LHCII amplified by PCR. In red the restriction site added during the cloning procedure.
(DOCX)
