# Supplementary figures and images for: Characterization of the Major Light-Harvesting Complexes (LHCBM) of the Green Alga Chlamydomonas reinhardtii
Source: PLoS One. 2015 Feb 27;10(2):e0119211. doi: 10.1371/journal.pone.0119211 (PMC4344250; doi:10.1371/journal.pone.0119211)

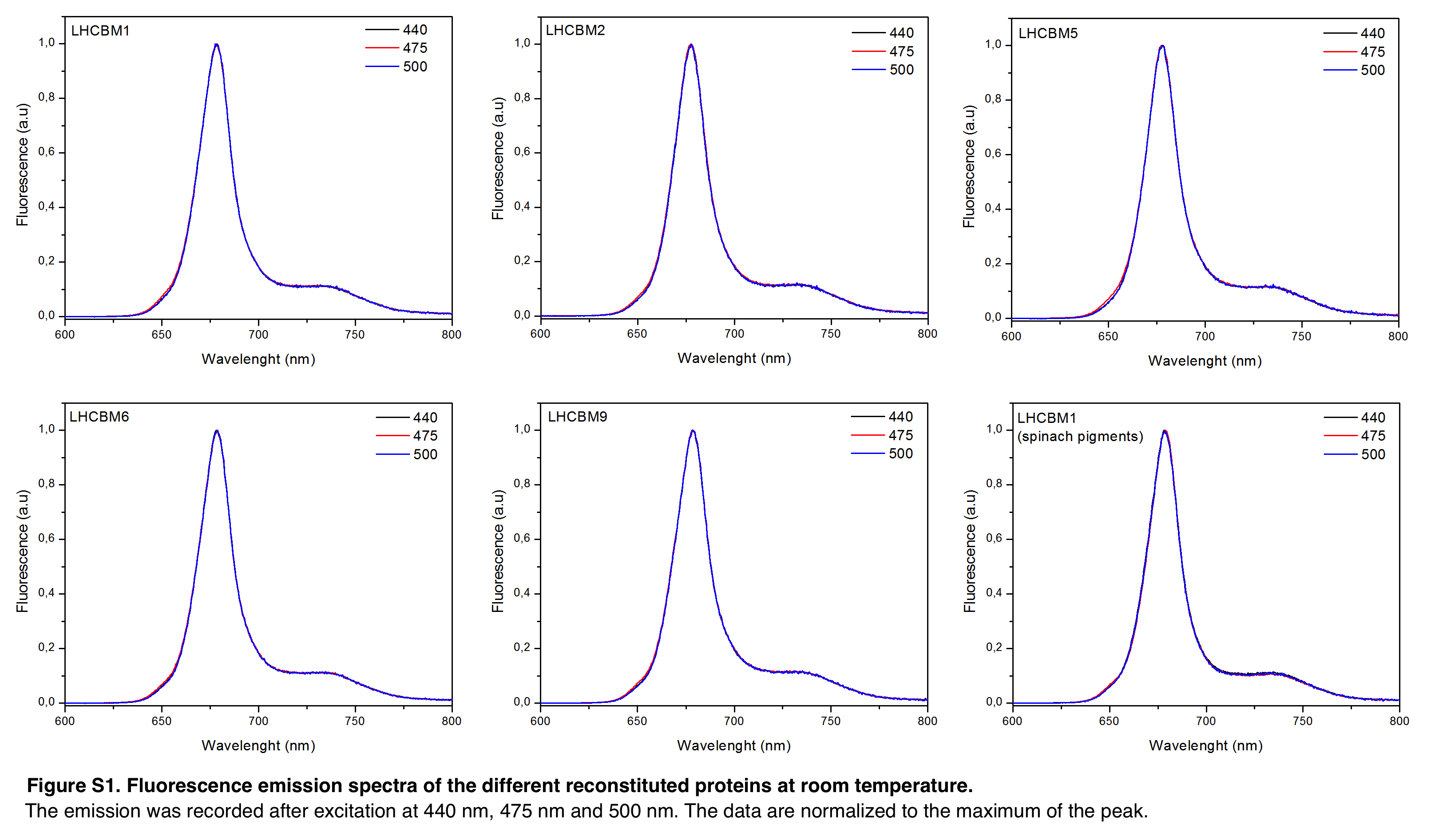

Supplement: S1 Fig — The emission was recorded after excitation at 440 nm, 475 nm and 500 nm. The data are normalized to the maximum. (TIF) [file pone.0119211.s001.tif]

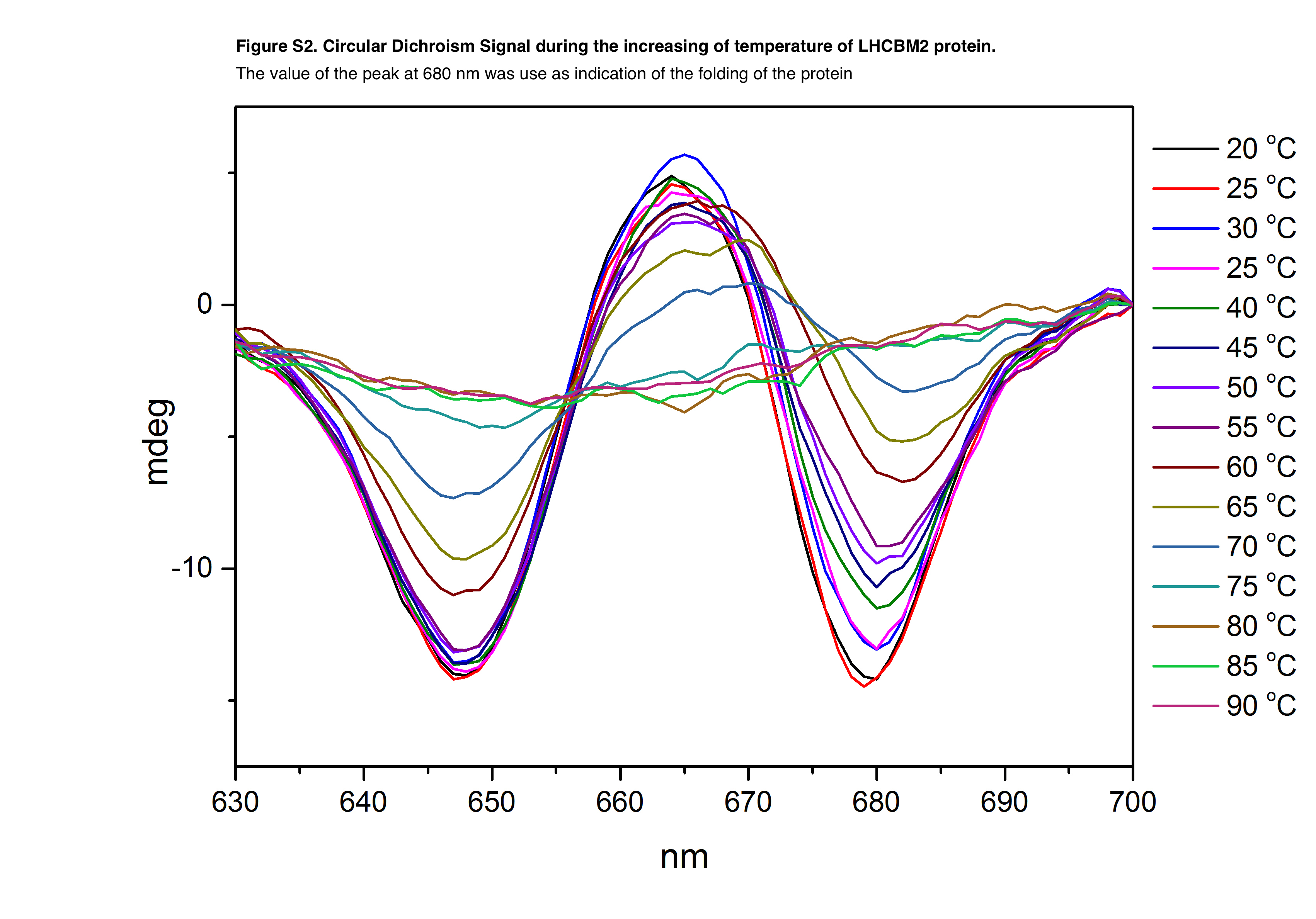

Supplement: S2 Fig — (JPG) [file pone.0119211.s002.jpg]
