# Supplementary material for: Characterization of the Major Light-Harvesting Complexes (LHCBM) of the Green Alga Chlamydomonas reinhardtii
Source: PLoS One. 2015 Feb 27;10(2):e0119211. doi: 10.1371/journal.pone.0119211 (PMC4344250; doi:10.1371/journal.pone.0119211)
Supplement: S1 Table — In red the restriction site added during the cloning procedure. (DOCX) [file pone.0119211.s003.docx]

| **gene name** | **Forward primer** | **Reverse primer** | **Restriction site** |
| --- | --- | --- | --- |
| *LHCBM1* | tGAATTCGCCTTCGCTCTGGCGT | attGTCGACGGCCGAGGGGGTGAA | EcoRI/SalI |
| *LHCBM2* | attGGATCCGCCGCCATCATGAAGTCCGCT | ataaCTCGAGGGCCGAGGGGGTGAACTTG | BamHI/XhoI |
| *LHCBM5* | attGGATCCATGATGCTGTCTCGCACCGT | ataaCTCGAGCTGAGGGGTGAACTTCTGG | BamHI/XhoI |
| *LHCBM6* | tGAATTCGCCTTCGCTCTGGCGT | attGTCGACGGCCGAGGGGGTGAA | EcoRI/SalI |
| *LHCBM9* | attGAATTCGCATTCGCTCTGGCCTC | ataaGTCGACCGCCGAGGGAGTGTA | EcoRI/SalI |

**Table S1. Primer list of the different LHCII amplified by PCR.** In red the restric,on site added during the cloning procedure.
